# Malignant Melanoma-Derived Exosomes Induce Endothelial Damage and Glial Activation on a Human BBB Chip Model

**DOI:** 10.3390/bios12020089

**Published:** 2022-01-31

**Authors:** Peng Wang, Yunsong Wu, Wenwen Chen, Min Zhang, Jianhua Qin

**Affiliations:** 1 Division of Biotechnology, Dalian Institute of Chemical Physics, Chinese Academy of Sciences, Dalian 116023, China; wangpeng1907@dicp.ac.cn (P.W.); wuyunsong@dicp.ac.cn (Y.W.); chenwenwen@dicp.ac.cn (W.C.); zhangmin@dicp.ac.cn (M.Z.); 2 School of Future Technology, University of Chinese Academy of Sciences, Beijing 116023, China; 3 Institute for Stem Cell and Regeneration, Chinese Academy of Sciences, Beijing 100864, China; 4 CAS Center for Excellence in Brain Science and Intelligence Technology, Chinese Academy of Sciences, Shanghai 200031, China

**Keywords:** blood–brain barrier (BBB), organ chip, brain metastasis, malignant melanoma, exosomes

## Abstract

Malignant melanoma is a type of highly aggressive tumor, which has a strong ability to metastasize to brain, and 60–70% of patients die from the spread of the tumor into the central nervous system. Exosomes are a type of nano-sized vesicle secreted by most living cells, and accumulated studies have reported that they play crucial roles in brain tumor metastasis, such as breast cancer and lung cancer. However, it is unclear whether exosomes also participate in the brain metastasis of malignant melanoma. Here, we established a human blood–brain barrier (BBB) model by co-culturing human brain microvascular endothelial cells, astrocytes and microglial cells under a biomimetic condition, and used this model to explore the potential roles of exosomes derived from malignant melanoma in modulating BBB integrity. Our findings showed that malignant melanoma-derived exosomes disrupted BBB integrity and induced glial activation on the BBB chip. Transcriptome analyses revealed dys-regulation of autophagy and immune responses following tumor exosome treatment. These studies indicated malignant melanoma cells might modulate BBB integrity via exosomes, and verified the feasibility of a BBB chip as an ideal platform for studies of brain metastasis of tumors in vitro.

## 1. Introduction

Malignant melanoma is a type of highly malignant tumor, derived from melanocytes, that mainly occurs in skin, mucosa and innards [1,2]. In recent years, the morbidity and fatality of malignant melanoma has continued to rise [2]; however, there is still no effective treatment except resection surgery for this devastating disease. Malignant melanoma has a high metastasis ability, and the brain is the most common metastasis site [3]. About 70% of malignant melanoma patients die from the diffusion of tumor cells in the central nervous system (CNS) [4]. To date, the early diagnosis and treatment of malignant melanoma brain metastasis is still a great challenge.

In the human brain, there is a protective barrier system between blood circulation and human brain parenchyma, the blood–brain barrier (BBB), which consists of brain microvascular endothelial cells, basal lamina, perivascular astrocytic end feet and pericytes [5,6]. The BBB is a dynamic and selective interface, which is responsible for regulating the passage of nutrients and metabolic waste in human brains [7,8]. Moreover, the BBB functions in protecting the brain from the invasion of mostly toxic substances, including tumor cells. It is still unclear how a malignant melanoma crosses the BBB and achieves brain metastasis.

Exosomes are a type of nano-sized vesicle (30–150 nm diameter) secreted by most living cells [9,10]. They are enclosed by a lipid bilayer and load various biomacromolecules, such as nucleic acids and proteins. Exosomes are widely distributed in a variety of bodily fluids. Recent studies have reported that exosomes play crucial roles in mediating brain tumor metastasis, such as breast cancer and lung cancer. Some studies have reported that exosomes derived from metastatic cancer cells can modulate the microenvironment of neurovascular unit (e.g., glucose metabolism level or microglial state) in a premetastatic niche to promote metastasis [11,12]. Moreover, recent studies have revealed that exosomes derived from metastatic cancer cells can act directly on vascular endothelial cells, disrupt endothelial integrity, and increase the permeability of the BBB using non-coding RNAs (including microRNA and lncRNA) [13,14,15]. However, little is known about whether exosomes derived from melanoma cells have similar effects on the BBB or neurovascular unit in humans.

At present, one major challenge for studies of brain tumor metastasis lies in the lack of suitable BBB models for tumor cell migration. Classical animal models, such as rodents, have been widely used [16,17,18,19,20]. However, differences in genetics and physiology make them difficult in replication of pathological changes in humans. Regarding in vitro models, a co-culture system on a transwell is a common method [13,21,22,23], while the lack of physiologically relevant factors, such as fluidic flow and extracellular matrix environment, limits its application. In the past decade, organ-on-chip (OOC) technology has gained significant development, as it can reconstruct 3D units in vitro and replicate the key functions from native human organs or tissues. OOC have been applied in the building of various in vitro tissues and widely used for disease modeling and drug screening [24,25,26,27,28]. In recent years, many BBB chip models have been developed that have allowed co-culture of various types of cells under microfluidic conditions, and were widely used in the simulation of BBB functions, the modeling of neurological diseases, and drug transport testing [27,29,30,31,32]. Compared with traditional co-culture systems based on transwell and animal models, BBB chips can mimic in vitro physiological functions of human BBB and intercellular interactions between distinct cell types in a more physiology-relevant manner; moreover, they provide an optically clear window to observe biological responses in the BBB in a real-time manner.

In this study, we built a human BBB chip by co-culturing brain microvascular endothelial cells, astrocytes, and microglial cells under microfluidic conditions, and used it to test the effects of tumor-derived exosomes on the human BBB. The results indicated malignant melanoma cells might modulate BBB integrity and remodel brain microenvironment via exosomes, which will expand our understanding of the molecular basis underlying brain metastasis of the tumor.

## 2. Materials and Methods

### 2.1. Fabrication of the BBB-on-a-Chip System

In this study, the BBB-on-a-chip device was fabricated with PDMS (Dow Corning, SYLGARD 184) using technology. In the beginning, SU-8 3025 negative photoresistor was poured onto the glass to a height of 200 μm and degassed. It was allowed to selectively crosslink under UV light as it was covered by the mask. After excessive SU-8 was removed, the template was patterned. The BBB chip consists of PDMS layers, a top endothelial channel, and a bottom glial channel. Two layers were fabricated with the same template and separated with a PET porous film. PDMS layer was created by pouring PDMS pre-polymer containing a 10:1 (wt./wt.) mixture of elastomer base to curing agent. PDMS pre-polymer was polymerized in an oven at 80 °C for 1 h, then inlet and outlet holes in the upper layer were formed with a 0.8-mm diameter sharp needle. The PDMS layer contained a channel as a culture chamber (width: 1.0 mm; height: 0.2 mm). Next, a PET porous film was sandwiched and bonded between the top and bottom PDMS layers after a 20 s oxygen plasma treatment (power: 250 W). Then, the device was placed in an oven at 80 °C for 2 h and kept sterile until use.

The assembled microfluidic chip was exposed to UV light overnight in biosafety cabinet for sterilization. Prior to cell seeding, both channels were pre-coated with 0.2% collagen type I at 37 °C overnight. HA cells and HMC3 cells which were mixed in a 3:1 ratio, were seeded at 3 × 10^6^ cells/mL density into the glial channel and incubated for 2 h to allow the adhesion of cells onto the porous membrane while the chip was placed upside down. Then, the hCMEC/D3 cells were seeded into the endothelial channel with the density of a 4 × 10^6^ cells/mL. One day later, cells on the BBB chip were stabilized, and the endothelial channel and glial channel were connected with syringe pump (LongerPump, #LSP10-1B) which provides a continuous flow of medium with a flow rate of 100 μL/h. The hCMEC/D3 cells in endothelial channel were cultured in ECM medium (ScienCell, #1001), and the HA cells and HMC3 cells in the glial channel were cultured in a mixed medium at a 1:1 ratio of AM medium (ScienCell, #1801) and MEM medium (Procell, #PM150410) (supplemented with 10% FBS).

### 2.2. Cell Culture

hCMEC/D3 cells (iCell, hCMEC/D3) were cultured in ECM medium (ScienCell, #1001). HA cells (ScienCell, #1800) were cultured in AM medium (ScienCell, #1801). HMC3 cells (Procell, #HMC3) were cultured in MEM medium (Procell, #PM150410) supplemented with 10% FBS (Gibco, #10099-141) and 1% P/S (Gibco, #15140-122). A375 cells (iCell, #A375) were cultured in DMEM medium (Gibco, #C11995500BT) supplemented with 10% FBS and 1% P/S. A549 cells (iCell, #A549) were cultured in DMEM medium supplemented with 10% FBS and 1% P/S. Caco-2 cells (iCell, #Caco-2) were cultured in DMEM medium supplemented with 10% FBS and 1% P/S. MDA-MB-231 cells (iCell, MDA-MB-231) were cultured in RMPI-1640 medium (Gibco, #C11875500BT) supplemented with 10% FBS and 1% P/S.

### 2.3. Exosome Purification

To isolate exosomes from the cell culture medium, exosomes in fetal bovine serum (FBS) were removed first. FBS was centrifuged at 1000× *g* for 15 min, then 10,000× *g* for 20 min at 4 °C, then the supernatant was filtered with a 0.22-μm filter. Next, the serum was centrifuged at 120,000× *g* for 12 h at 4 °C to precipitate exosomes. Tumor cells were cultured in the medium containing exosome-free FBS for 48 h.

To purify exosomes from the culture medium, the collected medium was centrifuged at 1000× *g* for 10 min, then 10,000× *g* for 30 min at 4 °C to precipitate cells and cell debris, then the supernatant was filtered with a 0.22-μm filter. Next, exosomes were centrifuged at 120,000× *g* for 70 min at 4 °C. Exosomes were washed with PBS, collected by ultracentrifugation at 120,000× *g* for 70 min at 4 °C and finally resuspended with PBS.

Exosome quantifications were determined using a fluorometer (Qubit 3.0, Thermo Fisher Scientific, Waltham, MA, USA) and protein assay kit (Invitrogen, Waltham, MA, USA). In brief, the standard protein samples with different concentrations (0, 200, 400 μg/mL) were used to set up a protein standard curve. Then the concentration of exosomes could be quantified using the form of relative protein content.

### 2.4. Exosome Staining with PKH67

Exosomes were stained with PKH67 Fluorescent Cell Linker Kits (Sigma-Aldrich, #MIDI67-1KT, St. Louis, MO, USA) according to the manufacturer protocol. Briefly, the purified exosomes were resuspended in PBS, and then immediately mixed with equal volume of diluent C which contained 4 × 10^−6^ M PKH67 dye. The mixture was incubated for 5 min before equal volume of 1% BSA was added to stop the staining. Next, the samples were centrifuged at 150,000× *g* for 1 h at 4 °C to remove excess dye, and the exosomes were resuspended in PBS.

PKH67-labeled exosomes were incubated with cells at 37 °C for 24 h. At the end of incubation, cells were washed in PBS three times and fixed with 4% paraformaldehyde for imaging analysis.

### 2.5. Exosome Detection by Transmission Electron Microscope

Exosomes were purified from tumor cells as described above, and resuspended in PBS. Before staining, exosomes were fixed in 4% paraformaldehyde for 30 min at 4 °C. Exosomes were loaded on the copper grid for 5 min, and the excess liquid was removed with filter paper. The exosomes were stained with uranyl acetate for 2 min. Then, the grid was dried at 85 °C and examined under a transmission electron microscope (TEM).

### 2.6. IL-6 Detection by ELISA Kit

The level of IL-6 was measured by Human IL-6 ELISA Kit (Invitrogen, #BMS213-2) according to the manufacturer protocol.

### 2.7. Immunostaining

Cells were fixed with 4% paraformaldehyde for 30 min and washed with phosphate-buffered saline (PBS; meilunbio, #MA0015, Dalian, China) three times for 5 min. The samples were incubated with 0.2% Triton-X 100 for 10 min and the cells were blocked in 5% normal goat serum for 30 min. Next, cells were incubated with primary antibodies overnight at 4 °C, and were then incubated with secondary antibodies at room temperature for 1 h. After staining with antibodies, cells were counterstained with DAPI before mounting (ThermoFisher, #P36980, Waltham, MA, USA). Images were acquired using an Olympus FV1000 confocal fluorescent microscope system.

### 2.8. RNA Extraction, Library Preparation and Sequencing

50 μL TRIzol reagent (Invitrogen) was infused into the upper endothelial channel and lower glial channel of the chip to lyse the cells for 30 s, and the lysates of hCMEC/D3 and glial cells (HA cells and HMC3 cells) were collected separately for RNA extraction. DNA digestion was carried out after RNA extraction by DNaseI. RNA quality was determined by examining A260/A280 using NanodropTM OneCspectrophotometer (Thermo Fisher Scientific Inc., Hongkong, China). RNA integrity was verified by 1.5% agarose gel electrophoresis. Qualified RNAs were finally quantified by Qubit 3.0 with QubitTM RNA Broad Range Assay kit (Life Technologies, Carlsbad, CA, USA). A total of 500 ng total RNAs were used for stranded RNA sequencing library preparation using KC-DigitalTM Stranded mRNA Library Prep Kit for Illumina^®^ (Catalog NO. DR08502, Wuhan SeqHealth Co., Ltd., Wuhan, China) following the manufacturer instruction. The kit eliminates duplication bias in PCR and sequencing steps, using unique molecular identifier (UMI) of 8 random bases to label the pre-amplified cDNA molecules. The library products corresponding to 200–500 bps were enriched, quantified, and finally sequenced on Hiseq X 10 sequencer (Illumina, San Diego, CA, USA).

### 2.9. RNA-Seq Data Analysis

Raw sequencing data were loaded into Trimmomatic (version 0.36) to filter low-quality reads and trim adaptors. Clean reads were further treated with in-house scripts to eliminate duplication bias introduced in library preparation and sequencing. In brief, adaptor-trimmed reads were first clustered according to the UMI sequences, in which reads with the same UMI sequence were grouped into the same cluster, resulting in 65,536 clusters. Reads with sequence identity over 95% in the same cluster were further extracted to a new sub-cluster using a reciprocal pairwise alignment strategy. After all sub-clusters were generated, multiple sequence alignment was performed to obtain one consensus sequence for each sub-cluster. After these steps, any errors and biases introduced by PCR amplification or sequencing were eliminated.

The de-duplicated clean reads were used for following RNA-seq analysis. First, the clean reads were mapped to the human reference genome (Ensembl database (ftp://ftp.ensembl.org/pub/release-87/fasta/homo_sapiens/dna/) (accessed on 8 December 2016)) using STAR software (version 2.5.3a) with default parameters. FeatureCounts (Subread-1.5.1) was then used to do quantification and RPKMs were calculated. Differentially expressed genes between the control group and A375 cell exosomes-treated group were identified using the edgeR package (version 3.12.1). An FDR corrected *p*-value < 0.05 and fold-change ≥ 1.5 were used to judge the statistical significance of gene expression differences. Gene ontology (GO) enrichment analysis for differentially expressed genes was implemented by KOBAS software (version: 2.1.1) with a corrected *p*-value < 0.05 to judge whether it has statistically significant enrichment.

### 2.10. Permeability Assay Using FITC-Dextran

A total of 0.2 mg/mL FITC-dextran (10 kDa) in ECM medium was infused into the upper vascular channel of the control or exosome-treated BBB chips. A total of 2 h later, the medium in the lower glial channel was collected and measured by a microplate reader at 488 nm (excitation) and 530 nm (emission) for FITC-dextran concentration determination.

The apparent permeability coefficient (*P_app_*) was calculated according to the equation below:

$$P_{app}=\frac{V_{g}C_{g}}{AC_{en}t}$$

where *V_g_* (mL) and *C_g_* (mol/mL) indicate the volume and concentration of FITC-dextran in the glial channel, respectively; *A* (cm^2^) indicates the contact area between endothelial channel and glial channel; *C_en_* (mol/mL) is the FITC-dextran concentration in the endothelial channel; and *t* (s) is the diffusion time of FITC-dextran.

### 2.11. Western Blot

For exosome detection, exosome lysate samples were separated on 10% SDS-PAGE and then transferred onto 0.2-μm nitrocellulose membranes. After being blocked with 0.5% skimmed milk powder in TBST buffer containing 0.05% Tween-20, the membranes were probed with the anti-CD63 antibody (Bioworld, BS72936) and anti-CD81 antibody (Proteintech, 66866-1-Ig) at 4 °C overnight, respectively. After that, the membranes were probed with corresponding horseradish peroxidase (HRP)-conjugated secondary antibodies at room temperature for 1 h at room temperature. Protein bands were detected by Prime Western Blotting Detection Reagent (GE Life, Singapore).

For the detection of brain endothelial cells, cell samples were separated on 10% SDS-PAGE and then transferred onto 0.2-μm nitrocellulose membranes. After being blocked with 0.5% skimmed milk powder in TBST buffer containing 0.05% Tween-20, the membranes were probed with the anti-ZO-1 antibody (Abcam, Ab96587), anti-VE-cadherin antibody (Proteintech, 66804-1-Ig) and anti-GAPDH (CWBIO, CW0100) at 4 °C overnight, respectively. After that, the membranes were probed with corresponding horseradish peroxidase (HRP)-conjugated secondary antibodies at room temperature for 1 h at room temperature. Protein bands were detected by Prime Western Blotting Detection Reagent (GE Life).

### 2.12. Antibodies for Immunostaining and Lab Consumables

Anti-IBA1 antibodies (10904-1-AP; 66827-1-Ig), anti-GFAP antibody (16825-1-AP) and anti-VE-cadherin antibody (66804-1-Ig) were purchased from Proteintech Group. Anti-Claudin-5 antibody (YTO952) was purchased from ImmunoWay. Anti-GFAP antibody (EM140707) was purchased from HUABIO. Anti-ZO-1 antibody (Ab96587) was purchased from Abcam. 

All secondary antibodies were purchased from Abcam group (anti-mouse Alexa Fluor 488, ab150113; anti-rabbit Alexa Fluor 488, ab150077; anti-mouse Alexa Fluor 594, ab150116; anti-rabbit Alexa Fluor 594, ab150080).

Lab consumables were bought from Guangzhou Jet Bio-Filtration Co., Ltd (Guangzhou, China).

### 2.13. Statistical Analyses

Data were analyzed by GraphPad Prism 6 software. Differences between two groups were analyzed using an unpaired Student’s *t*-test. Multiple group comparisons were performed using a one-way analysis of variance (ANOVA) followed by Bonferroni post hoc tests. Data were represented as mean ± standard error of the mean (SEM). Significance is indicated by asterisks: * *p* < 0.05; ** *p* < 0.01; *** *p* < 0.001.

## 3. Results

### 3.1. Exosomes Derived from Malignant Melanoma Cells Were Absorbed More Easily by Human Brain Microvascular Endothelial Cells

**Figure 1:**
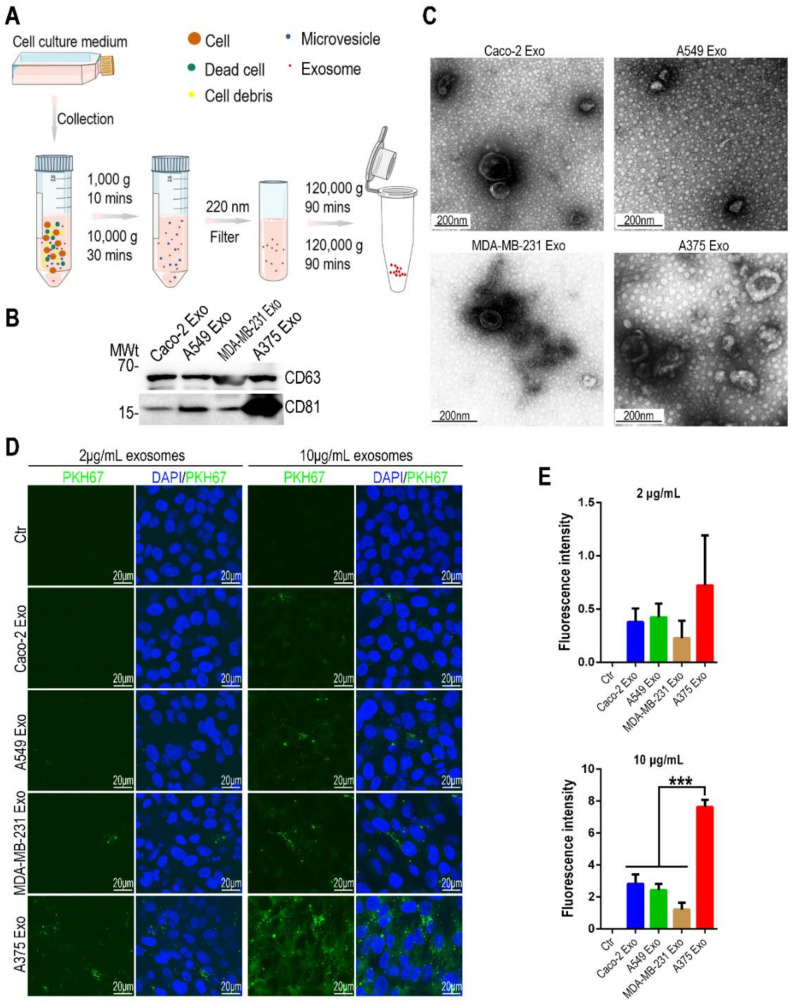
Exosomes derived from A375 cells were more easily absorbed by human brain microvascular endothelial cells. (**A**) Schematic diagram of exosomes purification from tumor cells by differential centrifugation. (**B**) Western blotting showing protein level of exosomal markers (CD63, CD81) in 4 types of tumor cell-derived exosomes (*n* = 3). (**C**) Representative TEM images of 4 types of tumor cell-derived exosomes (*n* = 2). (**D**) Representative confocal images of hCMEC/D3 cells 24 h after incubation with exosomes (2 μg/mL and 10 μg/mL) derived from 4 different types of tumor cells (*n* = 4). Exosomes were labeled by PKH67 (green) fluorescent dye. (**E**) Quantification of PKH67 fluorescence intensity for each group based on (**D**) (*n* = 4). Data are presented as mean ± SEM, and are analyzed using a one-way ANOVA with Bonferroni post-test (***: *p* < 0.001).

Initially, we purified exosomes from 4 types of common tumors cell lines (including Caco-2 colorectal cancer cell, A549 lung cancer cell, MDA-MB-231 breast cancer cell and A375 malignant melanoma cell) by means of ultracentrifugation (Figure 1A). According to clinical studies, these 4 types of cancers account for the majority of brain metastasis in patients [33].

The exosome samples were first detected by Western blotting for exosomal markers, and the results showed all 4 types of tumor cell-derived exosomes expressed CD63 and CD81 (Figure 1B). Moreover, the exosome samples were determined by transmission electron microscope (TEM), and particles showing lipid bilayer structure with a size range from 30–200 nm were observed (Figure 1C). This finding is consistent with previous reports [34,35,36,37]. The 4 types of tumor cell-derived exosomes were labeled with fluorescent dye (PKH67), and diluted in the ECM medium (2 μg/mL and 10 μg/mL). Then, the labeled exosomes were incubated with human brain microvascular endothelial cells (hCMEC/D3) for exosome uptake analysis. A total of 24 h later, confocal microscopic analysis showed the brain microvascular endothelial cells absorbed the exosomes derived from A375 cells at the highest level among the 4 types of exosomes (Figure 1D). Moreover, we quantified the uptake efficacy for exosomes based on fluorescent intensity, and the results further verified exosomes derived from malignant melanoma cells were more easily absorbed by human microvascular endothelial cells (Figure 1E).

### 3.2. Malignant Melanoma Exosomes Could Penetrate the BBB and Affect Its Function on the Organ Chip

Brain microvascular endothelial cells are the core component of the BBB. Since melanoma cell-derived exosomes easily entered the human microvascular endothelial cells, we then hypothesized whether they can penetrate the BBB and affect its function.

**Figure 2:**
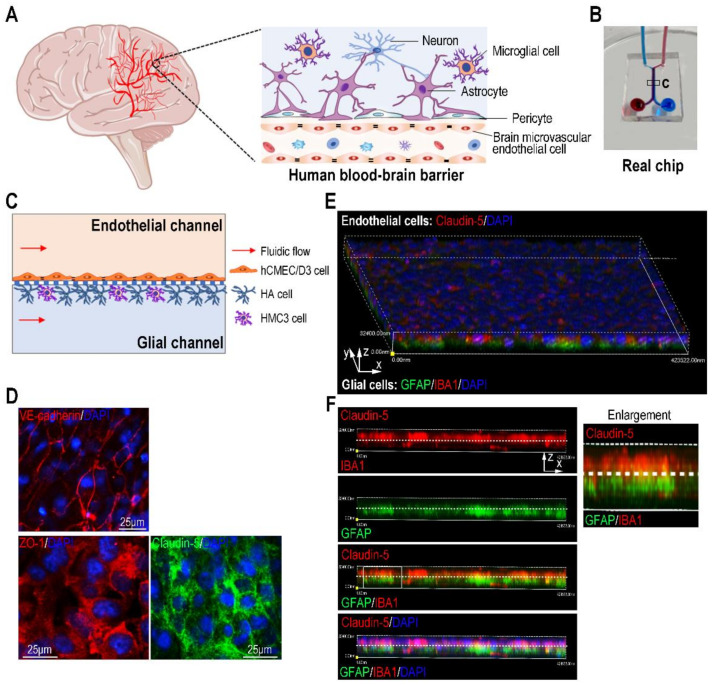
Establishing a human BBB chip. (**A**) A schematic description of human brain and blood–brain barrier (BBB). (**B**) A real image of a BBB chip. Red and blue inks were poured into two channels. (**C**) Schematic description of the BBB chip by co-culturing of human brain microvascular endothelial cells, astrocytes and microglial cells on the porous PET membrane. (**D**) Representative confocal images of brain endothelial cells immunostained for VE-cadherin, ZO-1 and Claudin-5 on chip device under flow culture condition for 3 days (*n* = 3). (**E**) A 3D confocal image showed the BBB barrier interface on chip device. The BBB barrier interface was formed by co-culture of human brain microvascular endothelial cells (hCMEC/D3 cells; Clauding-5 staining), human astrocytes (HA cells; GFAP staining) and microglial cells (HMC3 cells, IBA1 staining) on the porous membrane under fluid flow conditions (*n* = 3). (**F**) Side views of the BBB interface identified by Claudin-5 in human brain microvascular endothelial cells, GFAP in astrocytes and IBA1 in microglial cells (*n* = 3). The area indicated by the white box was enlarged on the right. The white dotted line indicated the porous membrane.

To mimic the human BBB in vivo (Figure 2A), we constructed a 3D human BBB model based on organ-chip technology. The BBB chip comprised an endothelial channel and a glial channel separated by a porous PET (polyethylene terephthalate) membrane (10 μm thick, 2 μm diameter) (Figure 2B). hCMEC/D3 cells (human microvascular endothelial cells) were seeded on the upper surface of the porous membrane, and astrocytes (HA cells) and microglial cells (HMC3 cells) were seeded on the opposite surface of the membrane (Figure 2C). The BBB chip was cultured with 100 μL/h (equivalent to 0.0325 dyn/cm^2^) of continuous medium flow in both channels, as accumulated evidence indicated the expressions of tight junction proteins and some endothelial markers are flow-dependent, and a physiological shear stress promotes BBB maturation and function [29,38]. A total of 3 days later, confocal microscopic images revealed expressions of adherens junction VE-cadherin and tight junction proteins ZO-1 and Claudin-5 in brain endothelial cells (Figure 2D). Three-dimensional reconstruction imaging showed a BBB interface was formed on the porous membrane, identified by Claudin-5 expression (red) in brain endothelial cells, GFAP expression (green) in astrocytes and IBA1 expression (red) in microglial cells (Figure 2E,F).

**Figure 3:**
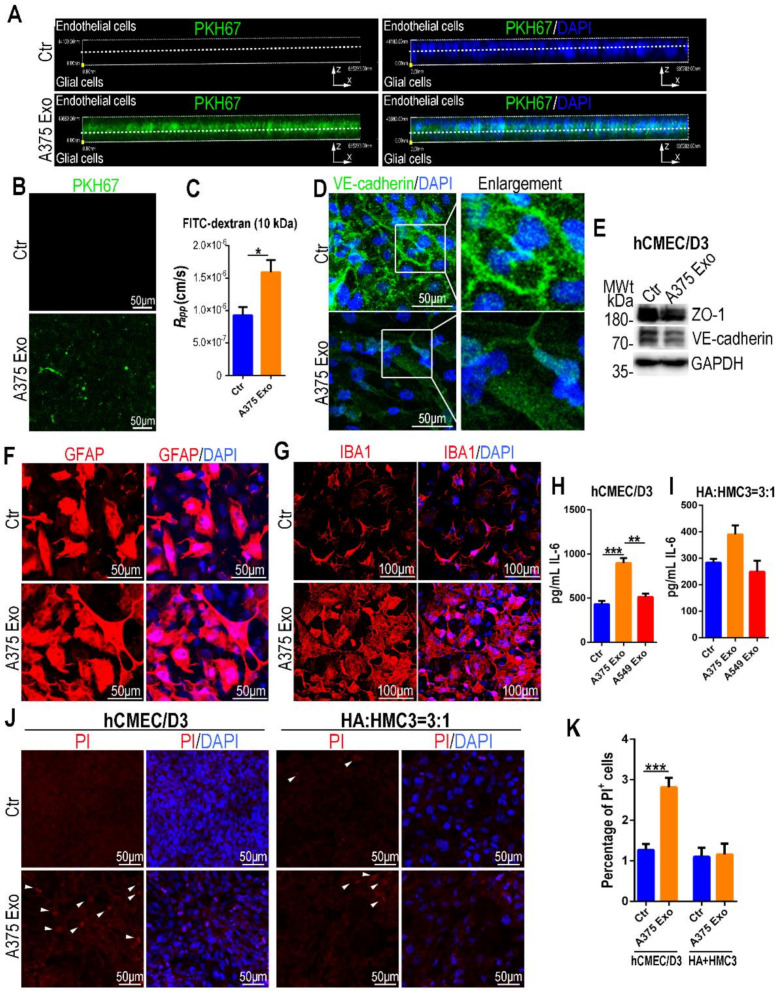
A375 cell-derived exosomes caused BBB damage and glial activation. (**A**) Side views of the BBB chip 24 h later following exposure of A375 cell-derived exosomes (green) to the vascular side (*n* = 3). (**B**) Representative confocal images of glial cells 24 h later following exposure of A375 cell-derived exosomes labeled by PKH67 dye to the vascular side (*n* = 3). (**C**) BBB permeability assay of 10 kDa FITC-dextran following exposure of A375 cell-derived exosomes to the vascular side (*n* = 4). Data are presented as mean ± SEM, and are analyzed using unpaired Student’s *t*-test (*: *p* < 0.05). (**D**) Representative confocal images of brain endothelial cells (VE-cadherin) 72 h later following treatment of A375 cell-derived exosomes (*n* = 3). The areas indicated by the white boxes were enlarged on the right. (**E**) Western blotting showing the protein level of VE-cadherin and ZO-1 in brain endothelial cells 72 h later following treatment of A375 cell-derived exosomes (*n* = 3). GAPDH was used as an internal control. (**F**,**G**) Representative confocal images of astrocytes (GFAP; **F**) and microglia (IBA1; **G**) 72 h later following treatment of A375 cell-derived exosomes (*n* = 3). (**H**) Bar graph showing IL-6 concentration in culture supernatant of vascular channel 72 h later following exposure of A375 or A549 cell-derived exosomes to the vascular side detected by ELISA kit (*n* = 3). (**I**) Bar graph showing IL-6 concentration in culture supernatant of glial channel 72 h later following exposure of A375 or A549 cell-derived exosomes to the vascular side detected by Elisa kit (*n* = 3). (**H**,**I**) Data are presented as mean ± SEM, and are analyzed using a one-way ANOVA with Bonferroni post-test (**: *p* < 0.01; ***: *p* < 0.001). (**J**) Representative confocal images of brain endothelial cells and glial cells stained with PI dye 72 h later following treatment of A375 cell-derived exosomes. Cells positive for PI were indicated by white arrowheads (*n* = 4). (**K**) Bar graph showing the percentage of PI^+^ cells for each group based on (**J**) (*n* = 4). Data are presented as mean ± SEM, and are analyzed using unpaired Student’s *t*-test (***: *p* < 0.001).

Next, we infused the ECM medium containing 10 μg/mL A375 cell-derived exosomes (pre-labeled with PKH67 dye) into the endothelial channel of the BBB chip. A total of 24 h later, confocal microscopic images showed that the exosomes penetrated the BBB interface (Figure 3A) and were absorbed by cells in glial channel (Figure 3B). Further examination revealed an increase of BBB permeability using FITC-dextran (10 kDa) from vasculature to the glial side 72 h later, following exposure of A375 cell-derived exosomes (Figure 3C). At the same time, an obvious decrease of VE-cadherin (an adherens junction protein) in brain endothelial cells followed treatment of A375 cell-derived exosomes by confocal microscope (Figure 3D). Western blotting revealed a significant down-regulation of VE-cadherin and ZO-1 (a tight junction protein) in brain endothelial cells as well (Figure 3E). Moreover, confocal images of glial cells indicated treatment of A375 cell-derived exosomes induced up-regulation of GFAP in astrocytes (Figure 3F) and IBA1 in microglial cells (Figure 3G). Moreover, elevation of proinflammatory IL-6 was detected in both endothelial and glial channels, following treatment of A375 cell-derived exosomes. Treatment of A549 cell-derived exosomes had no visible effects on the BBB (Figure 3H,I). To test the effects of A375 cell-derived exosomes on the viability of both brain endothelial and glial cells, we also detected the cells by staining for PI dye. As Figure 3J,K showed, treatment of A375 cell-derived exosomes induced a significant increase of PI^+^ cells in brain endothelium, but had no obvious effect on glial cells. All these findings indicated malignant melanoma-derived exosomes could penetrate the BBB, and contributed to BBB disruption and glial activation.

### 3.3. Transcriptome Analysis of Brain Endothelial Cells and Glial Cells Following Treatment of Tumor Exosomes on BBB Chip

To gain a global view of the molecular basis underlying BBB dysfunction induced by A375 cell-derived exosomes, we performed transcriptome analysis of brain endothelial and glial cells on the BBB chip. Briefly, 3 days following treatment of A375 cell-derived exosomes, brain endothelial cells and glial cells (astrocytes and microglial cells) were collected, respectively, and analyzed by RNA sequencing.

**Figure 4:**
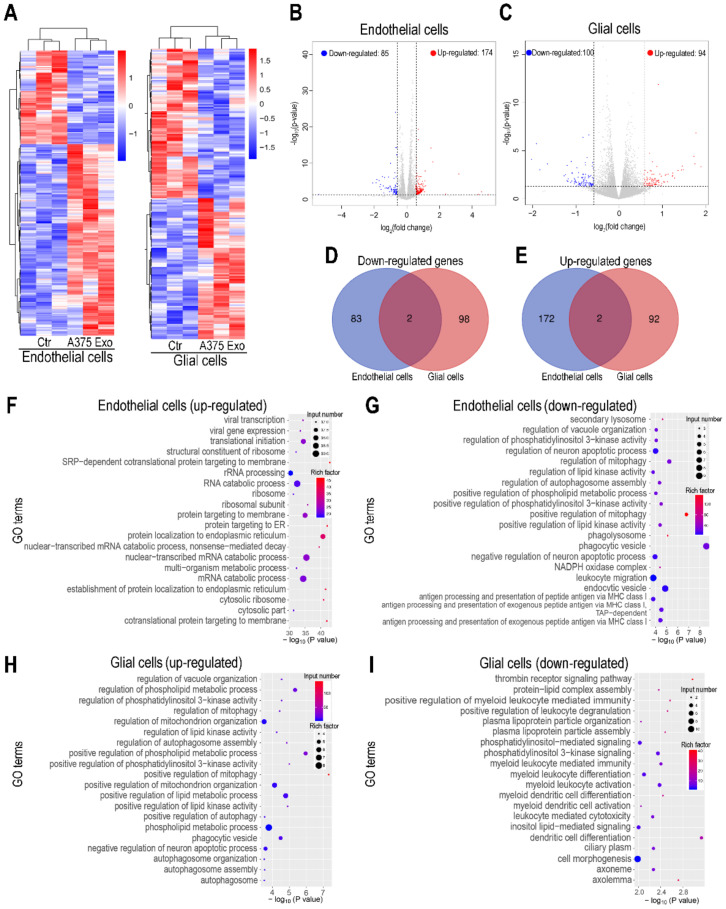
RNA-seq analysis of brain endothelial cells and glial cells following treatment of A375 cell-derived exosomes on the BBB chip. (**A**) Heat-map showing the transcriptional changes of brain endothelial cells and glial cells on BBB chip 3 days after treatment of A375 cell-derived exosomes (*n* = 3). (**B**,**C**) Volcano plots showed the modulated genes in brain endothelial cells (**B**) or glial cells (**C**) 3 days after treatment of A375 cell-derived exosomes. Genes differentially expressed with fold-change >1.5 and *p* < 0.05 were marked in color. *p*-values were calculated using a two-sided, unpaired Student’s t-test with equal variance assumed. (**D**) Venn diagrams depicted the down-regulated DEGs shared or unique between brain endothelial cells and glial cells. (**E**) Venn diagrams depicted the up-regulated DEGs shared or unique between brain endothelial cells and glial cells. (**F**,**G**) Dotplot showed the enriched GO terms based on up-regulated (**F**) or down-regulated (**G**) genes in brain endothelial cells following treatment of A375 cell-derived exosomes. (**H**,**I**) Dotplot showed the enriched GO terms based on up-regulated (**H**) or down-regulated (**I**) genes in glial cells following treatment of A375 cell-derived exosomes. (**F**–**I**) The color of the dots represents the rich factor, and the size represents the input number for each GO term.

A heat-map showed that treatment of A375 cell-derived exosomes induced transcriptome modulations in both brain endothelial and glial cells (Figure 4A). To identify the differentially expressed genes (DEGs), the cutoff values for the fold-change and *p*-value were set to 1.5 and 0.05, respectively. As a result, our analysis identified 259 and 194 differentially expressed genes (DEGs) in the brain endothelial cells and glial cells following treatment of A375 cell-derived exosomes (Figure 4B,C). Venn diagrams showed that only 4 DEGs (<1% of total DEGs) were shared between brain endothelial and glial cells, indicating A375 cell-derived exosomes affect brain endothelial cells and glial cells in different manners (Figure 4D,E).

To identify the modulated biological processes, we performed Gene Ontology (GO) analysis. The results showed, in the brain endothelial cells, that some up-regulated genes were particularly enriched in some RNA metabolisms related to GO terms, such as “RNA catabolic process”, “nuclear-transcribed mRNA catabolic process” and “mRNA catabolic process” (Figure 4F); and some down-regulated genes were enriched in some autophagy-regulated GO terms, such as “regulation of mitophagy” and “phagocytic vesicles” (Figure 4G). In glial cells, enriched GO terms included some autophagy-regulated biological processes such as “positive regulation of autophagy” and “autophagosome assembly” for up-regulated genes (Figure 4H); and immune-related biological processes such as “myeloid leukocyte-mediated immunity”, “myeloid leukocyte activation” (Figure 4I). Regarding the modulated genes in glial cells, we noticed some down-regulated genes were enriched in myeloid leukocyte activation and myeloid leukocyte-mediated immunity terms. Microglial cells are the resident immune cells in the nervous system derived from myeloid cells, which play a key role in monitoring the brain microenvironment [20,39,40]. The down-regulation of this biological processes may indicate the impairment of their surveillance function for the CNS environment [41].

## 4. Discussion

Malignant melanoma is a type of devastating tumor, with a strong ability to metastasize human brains. Recent studies have reported that exosomes contributed to brain metastasis of tumors, such as lung cancer and breast cancer [11,12,13,14,15]. Presently, it is unclear whether exosomes secreted by malignant melanoma cells are also involved in brain metastasis, especially regulating the BBB integrity.

In this study, we examined the absorbing ability of brain microvascular endothelial cells for various kinds of tumor exosomes, and it appeared that malignant melanoma-derived exosomes were more easily absorbed by the brain endothelial cells. Considering that human microvascular endothelial cells are the core component of BBB, we then speculated that malignant melanoma might modulate BBB integrity by regulating brain endothelial cells with exosomes. To test the hypothesis, we established a human BBB chip by co-culturing brain microvascular endothelial cells and glial cells, and used it to explore the effects of malignant melanoma-derived exosomes on BBB. We found that malignant melanoma-derived exosomes could penetrate the BBB interface and be absorbed by the glial cells in the brain side. Malignant melanoma-derived exosomes significantly down-regulated the expression of tight junction protein in brain endothelial cells and induced glial activation. Furthermore, RNA-seq data showed that malignant melanoma-derived exosomes modulated the processes of autophagy and RNA metabolism in endothelial cells and immune-related biological process in glial cells. All these findings suggested that malignant melanoma could modulate BBB function via exosomes.

In recent years, accumulated studies have offered evidence of exosome involvement in the brain metastasis of tumors. A study by Tominaga et al. reported breast cancer-derived exosomes caused BBB breakdown by exosomal microRNA [15]. In this study, they described that miR-181c carried by exosomes contributed to the abnormal localization of tight junction proteins (Claudin-5, Occludin and ZO-1) in endothelial cells [15]. Another study by Kinjyo et al. demonstrated that exosomes secreted by precursor B acute lymphoblastic leukemia (BCP-ALL) cells induced increased production of vascular endothelial growth factor-A (VEGF-AA) by astrocytes, which was related to the disruption of BBB integrity and leukemic invasion [42]. Regarding exosomes in lung cancer, Gan et al. reported lung tumor cell-derived exosomes regulated brain endothelial cells to secrete Dkk1and induced M2 phenotype conversion of microglia, which promote tumor cell colonization and proliferation in brains [11]. The role of exosomes in malignant melanoma brain metastasis has also attracted more attention in recent years. Kuroda et al. reported that CD46 is a major receptor for the uptake of melanoma exosomes by human brain microvascular endothelial cells [43]. Moreover, exosomes of various sizes were identified in melanoma interstitial space and several subpopulations of exosomes from human metastatic melanoma tissue were identified by quantitative proteomics [44]. It is noted that the most common preclinical models used in these studies are rodents. Due to model complexity and the species difference between the rodent and human systems, experimental controllability and reproducibility are undermined in clinical tests, which lead to high failure rates of new drugs in clinical trials [45]. The microfluidics-based organ-chip model can mimic the physiology and functionality of human organs on a chip, and it is hopeful that the gap between animal models and clinical trials can be bridged. Here, we made use of the BBB chip to establish the brain metastasis model, which provided a new method for the study of brain metastasis in vitro. Next, we will continue to explore whether the modulated BBB could promote malignant melanoma cells to access the brain parenchyma.

This work has some limitations. First, we only tested exosomes from one melanoma cell line (A375 cells), and additional melanoma cell lines are needed to test whether enhanced uptake for melanoma-derived exosomes by brain microvascular endothelial cells is a universal melanoma trait. Second, we only tested the melanoma-derived exosomes on BBB chips for a short time (3 days); the effects for a prolonged period (e.g., 2–4 weeks) remain to be seen. The study still lacks some quantitative indicators to reveal the changes of BBB permeability on the chip device, and more quantitative methods should be introduced in the following research, such as quantification of tight junction protein changes (e.g., Claudin-5, ZO-1), and values of transendothelial electrical resistance (TEER).

In summary, our study tried to investigate the effects of tumor-derived exosomes on human BBB using an organ-chip model. Our findings revealed the potential of exosomes derived from malignant melanoma in mediating BBB integrity and remodeling the brain microenvironment. These findings will deepen our understanding of the molecular basis underlying brain metastasis of malignant melanoma, and will also provide a platform for anti-tumor drug screening and testing.

## Data Availability

All relevant data are available in the manuscript. The raw data of RNA-seq have been deposited on Sequence Read Archive (SRA) under the accession number PRJNA779191.
